# Tumbleweed-inspired robots with hybrid mobility for terrestrial exploration

**DOI:** 10.1038/s41467-025-66513-1

**Published:** 2025-11-20

**Authors:** Sanjay Manoharan, Biruktait Lemecho, Mustafa M. Fadlelmula, Vivek Subramanian

**Affiliations:** Laboratory for Advanced Fabrication Technologies, Institute of Electrical and Micro Engineering, École Polytechnique Fédérale de Lausanne (EPFL), Neuchâtel, 2000 Switzerland

**Keywords:** Mechanical engineering, Engineering

## Abstract

We present a tumbleweed-inspired rover that integrates passive wind-driven mobility with selective active control, creating a hybrid system capable of carrying heavy payloads while consuming less energy. By analyzing airflow over natural tumbleweeds and solid spheres, we found that the porosity gradient and resulting wake dynamics in tumbleweeds substantially enhance aerodynamic drag and mobility relative to solid forms. Following these findings, we fabricated a bio-inspired spherical shell with a tumbleweed-mimicking porosity profile to enable passive, wind-driven locomotion. When passive motion stalls due to insufficient wind or obstacles, mobility was maintained through an embedded quadcopter, enabling active maneuvers like tumbling, spinning, gliding, and flying. This hybrid approach allows the system to remain primarily passive while activating propulsion only when necessary, reducing energy expenditure. Furthermore, we deployed a mesh network of multiple such rovers to generate spatially distributed environmental maps, with each unit functioning as both transmitter and receiver to ensure reliable data relay even as nodes drift apart. Extensive laboratory and field testing validated the applicability and effectiveness of this hybrid approach, establishing a foundation for unmanned, energy-efficient terrestrial exploration.

## Abstract (web summary)

Tumbleweeds harbor an aerodynamic secret: strategically placed holes that increase drag and enhance wind dispersal. In this study, the authors decoded this mechanism and engineered drones that harness wind to roll and switch to flight when needed.

## Introduction

**Fig. 1:**
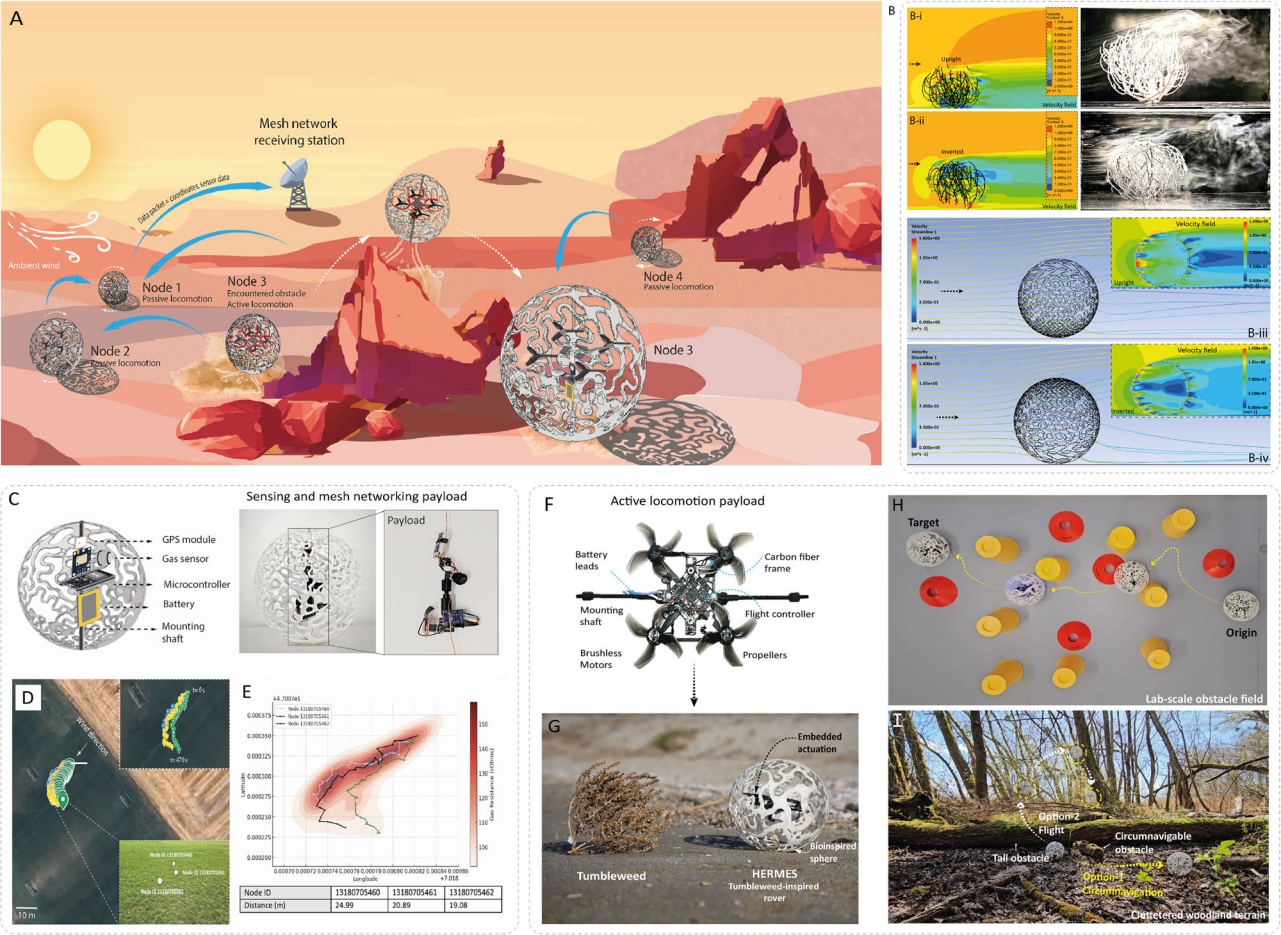
Graphical abstract of HERMES (Hybrid Energy-efficient Rover Mechanism for Exploration Systems) - a tumbleweed-inspired hybrid navigation rover. **A** Envisioned deployment of HERMES with passive rolling, active maneuvering for obstacle avoidance; and real-time spatiotemporal data transmission through a mesh network. **B** Flow dynamics of a natural tumbleweed in upright and inverted states, revealing novel drag-generation principles, which were subsequently applied to a bio-inspired sphere. **C** Sensor-equipped bio-inspired sphere with onboard GPS, microcontroller, battery, and environmental sensors, with inset showing the fabricated prototype. **D**, **E** Wind-driven trajectory mapping using GPS data and heatmap overlays of sensor readings along the path. **F**, **G** Embedded quadcopter for active control. **H** Active surface navigation to clear a lab-scale maze. **I** Field trial showing obstacle negotiation options - circumventing smaller barriers via surface navigation or taller obstacles by flying over.

Nature’s ingenious designs, refined over eons of evolutionary pressure, often offer simple solutions to complex challenges. Among these is the tumbleweed, a wind-dispersed plant capable of traversing vast, rugged terrain using ambient wind^1,2^. Despite erratic passive motion, tumbleweeds achieve efficient locomotion through subtle aerodynamic features largely unstudied in robotics. Unlike microfliers inspired from wind-dispersed seeds^3–5^, tumbleweeds travel farther and bear heavier loads^6^, making them an ideal terrestrial exploration model (Fig. 1A). While Argo floats^7^ and Radiosondes^8^ generate sensor maps by exploiting ocean and air current-induced motion, terrestrial sensing lacks equivalent systems^9^. Adapting tumbleweed-inspired motion to robotics could enable mobile sensor networks for Earth and beyond. This requires embracing wind as a propulsion source and rethinking drag, not as a hindrance, but as a means of locomotion.

Drag-driven robotics have primarily explored land sails, strandbeests^10,11^, and rolling inflatable spheres^12–16^, but these often require large form factors, complex deployment, and specialized control^17^. To generate sufficient drag, such systems scale up dramatically, limiting in-situ fabrication and deployment^18^. In nature, however, even small tumbleweeds demonstrate wind-driven movement, yet their aerodynamic behavior remains unstudied. Through computational fluid dynamics (CFD) and wind tunnel tests, we identified a previously unreported vertical porosity gradient, a structural asymmetry between the upper and lower hemispheres(Fig. 1B-i, ii), that alters wake dynamics and enhances pressure drag (Fig. 1B-iii, iv). This feature was then incorporated into bio-inspired spherical shells, enabling passive rolling at low wind speeds while carrying embedded electronics (Fig. 1C). Field tests showed that GPS and sensor-equipped spheres could autonomously disperse and transmit geotagged data via a decentralized Wi-Fi mesh^19^, demonstrating their potential for scalable, wind-driven sensing networks (Fig. 1D, E).

In nature, wind is a fickle ally; its randomness and terrain interactions often stall passive systems, limiting operational life^16^. In contrast, fully active systems circumvent these obstacles but at a high energetic cost. Hence, a hybrid approach offers a promising middle ground, using selective actuation to overcome stagnation or obstacles. To this end, prior systems used pendulum or motorized actuation within inflatables^20,21^. In contrast, we embed a quadcopter, leveraging the flux escape enabled by the porous shell for escaping stagnation, circumnavigating, or even flying over obstacles, while preserving predominantly passive navigation (Fig. 1F, G). Although drone-rover hybrids exist across research and commercial domains^22–30^, none are capable of passive locomotion^31^. While pendulum, flywheel, and hopping-based spherical systems exist^31,32^, none achieve the fusion of wind-driven and active locomotion. Our hybrid design fills this gap, enabling energy-efficient, large-area exploration.

In this study, we address three goals: (1) understanding tumbleweed aerodynamics, (2) designing a system for drag-based passive locomotion with heavy payloads at low wind speeds, and (3) developing a hybrid rover, HERMES (Hybrid Energy-efficient Rover Mechanism for Exploration Systems), combining passive wind-driven motion with active quadcopter-assisted actuation. Named after the Greek god of travelers and explorers, HERMES was validated through lab and field tests (Fig. 1H, I), demonstrating wind responsiveness, hybrid mobility, and reduced energy use. Its bio-inspired design enables scalable, energy-efficient exploration through rolling, gliding, and flight.

## Results

We worked to understand tumbleweed aerodynamics for passive motion, applying these principles to enable drag-based propulsion in a rover, and developing a hybrid rover that combines wind-driven passive motion with quadcopter-enabled active control.

### Tumbleweed aerodynamics

Although inflatable spheres appear suited for tumbleweed-like motion, calculations show they generate too little drag for compact robots (Supplementary Fig. 1A–E, Supp notes). Effective mobility would require impractically large surface area or extremely low mass. This limitation led us to investigate natural tumbleweeds more closely to uncover aerodynamic strategies viable for small-scale robotic systems.

**Fig. 2:**
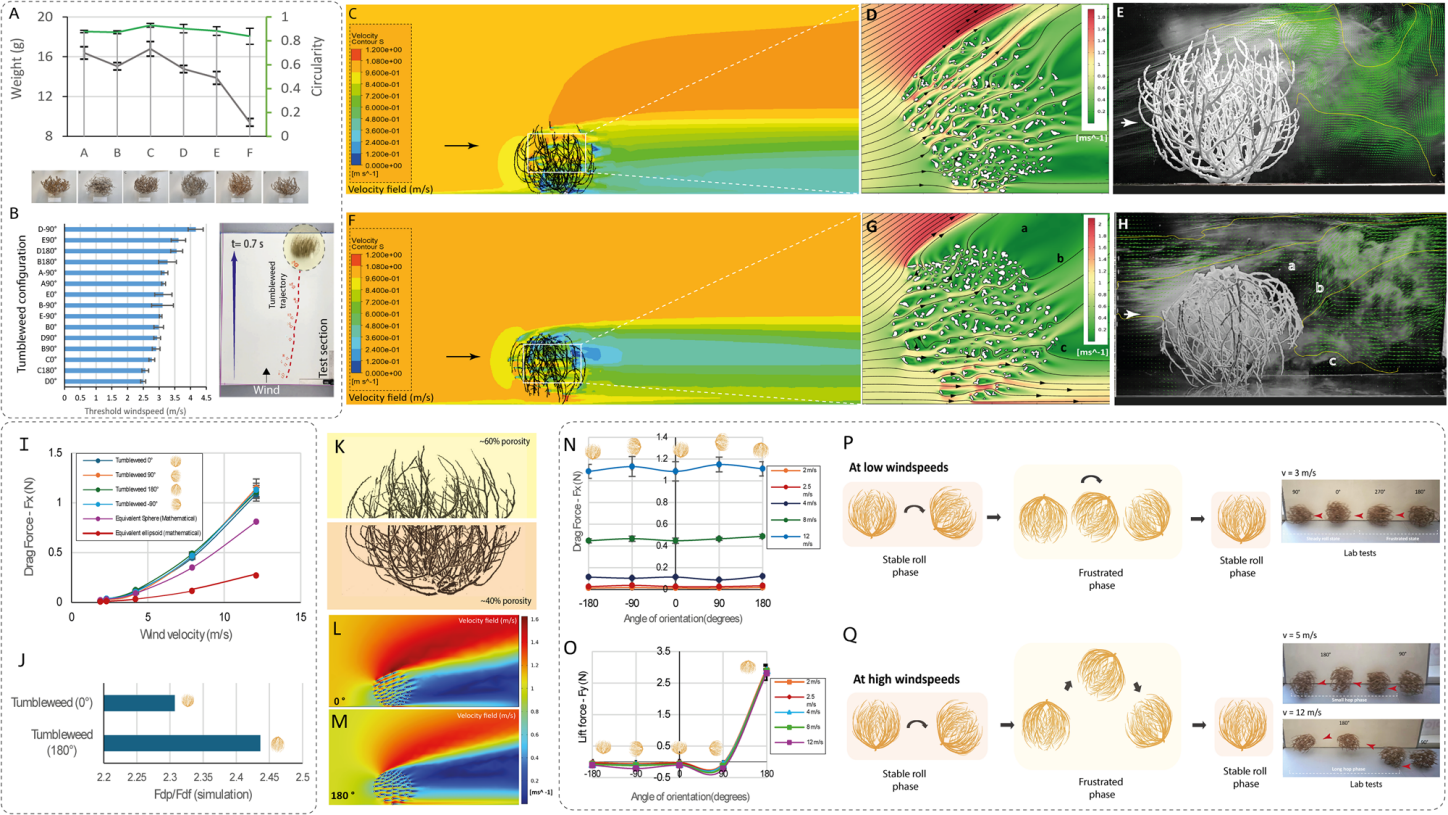
Aerodynamic characterization of natural tumbleweeds. **A** Mass and average circularity of six tumbleweed specimens (**A**–**F**). **B** Orientation-dependent threshold wind speeds for various specimens. **C**, **F** 3D CFD simulations showing velocity magnitude and streamlines for upright (0°) and inverted (180°) tumbleweed orientations under the same wind conditions. **D**, **G** 2D-CFD of the central cross-section showing flow divergence and wake structure, with the upright case producing a high-angle wake and the inverted configuration generating dual-lobed separation zones with stronger low pressure. **E**, **H** Wind tunnel smoke visualization confirming flow separation predicted by simulations. **I** Drag force measurements as a function of wind velocity (*n* = 3). **J** CFD-based pressure-to-skin-drag ratio confirming pressure drag dominance. **K** Optical porosity analysis showing a vertical porosity gradient. **L**, **M** 2D-CFD of porous circles mimicking the tumbleweed gradient. **N**, **O** Orientation-dependent drag and lift driving the pose transitions (*n* = 3). **P**, **Q** Locomotion models for low and high wind speeds. In the hop phase, elastic rebound lifts the tumbleweed, and the air gap enhances lift. The presence or absence of hop phase depends on branch compression, wind speed, and initial momentum.

To our knowledge, No prior studies have characterized the structural aerodynamic advantages of natural tumbleweeds. To explore this, we analyzed six tumbleweed specimens (*Salsola* genus, labeled A–F) to identify the most mobile. We prioritized mass and circularity, as both influence rolling resistance and wind responsiveness (Fig. 2A). Specimens B and D were the lightest, while C exhibited the highest circularity despite being slightly heavier. Since low mass enhances mobility and high circularity improves rolling efficiency, these characteristics guided our selection. Each specimen was tested in four orientations (0°, ±90°, 180°) under wind speeds ranging from 2 to 12 m/s (Fig. 2B). Video analysis showed that 0° (root-down) and 180° (root-up) allowed easier rolling, while lateral orientations increased drag. Although D rolled at the lowest wind speed, specimen C showed strong, consistent mobility in both favorable orientations. Its high circularity led to its selection for further study. It was then CT-scanned and SLS-printed in PA2200 for aerodynamic testing.

Wind tunnel tests showed tumbleweeds consistently produced more drag than solid spheres. At 12 m/s, tumbleweed drag reached ~1.2 N versus 0.8 N for a solid sphere (Fig. 2I). This trend held across all tested wind speeds. While porosity typically reduces drag by allowing airflow through, the tumbleweed’s internal branch arrangement creates complex wake interactions that amplify pressure drag, revealing an unexpected aerodynamic advantage in its branched, porous structure.

To understand this phenomenon, we performed 3D-CFD simulations and smoke flow visualization tests (Fig. 2C, F) and smoke flow visualization tests in a wind tunnel (Fig. 2E, H). These studies focused on the tumbleweed in two configurations: upright (0°) and flipped (180°). In the upright orientation, both techniques revealed a large wake angle and significant flow separation behind the tumbleweed, which are signatures of high-pressure drag. In contrast, the flipped orientation produced a dual-lobed wake and a clear low-pressure region, shifting the flow interaction. To understand the physical origins of these observations, we performed 2D-CFD simulations of the central tumbleweed slice, which uncovered a key drag factor: a hidden porosity gradient (Fig. 2D, G).

In the upright position, the upper half, being more porous, allowed airflow to pass through freely. In contrast, the lower half was denser and thus offered greater resistance. This porosity gradient caused streamlines to decelerate in the denser lower region while accelerating through the open upper region, as visualized in Fig. 2D. The resulting imbalance deflected air upward and outward, generating a high-angle wake. When the tumbleweed was flipped (180°), the gradient inverted. The denser region now at the top forced air to flow around its perimeter, mimicking a solid sphere (Fig.2G-a). Meanwhile, the lower, more porous portion allowed airflow to split into multiple exit routes, resulting in two distinct flow separation zones and the dual-lobed wake pattern observed in Fig.2G-b, c. The redirection of flow toward the ground likely creates a ground-effect-like phenomenon, reducing the normal force and increasing lift. As suspected earlier, simulations confirmed that pressure drag was the dominant component, as reflected in the pressure-to-skin drag ratio (Fdp/Fds) (Fig. 2J).

We additionally confirmed the porosity gradient through optical measurements of natural tumbleweeds (Fig. 2K). The top region exhibited ~60% porosity with thin, spaced branches, while the bottom had ~40% porosity and a denser structure. This gradient likely reflects a natural adaptation: lower branches provide anchoring, while upper branches, where seeds form, are lighter and detachable to aid dispersal. Similar gradients were observed in other specimens: A (71% → 57%), D (69% → 51%), and F (76% → 60%), confirming this as a consistent morphological feature across samples.

To evaluate its engineering applicability, we performed 2D-CFD simulations using porous circular structures with internal density gradients mimicking Tumbleweed C (Fig. 2L, M). The simulations reproduced key flow behaviors observed in real tumbleweeds: an upward-deflected wake in the upright orientation and dual-lobed separation in the inverted case. Airflow is again split into a perimeter stream and central exits, confirming that the porosity gradient alone induces distinct aerodynamic effects and complex wake behavior. These qualitative observations were translated into quantitative design rules via a refined drag model that relates the drag coefficient to the porosity gradient, defined as Δp = pt – pb, where pt and pb represent the top and bottom hemisphere porosities. The model follows: Cd(Δp) = a0 + a1Δp + a2(Δp)², with coefficients from CFD simulations. When pt ≠ pb, asymmetric wake separation increased drag; when pt ≈ pb, symmetric flow minimized it. This model guides the design of porous structures for stability and aerodynamic performance (Supplementary Fig. 2A, B; Notes S2).

To better understand the aerodynamic forces influencing tumbleweed locomotion, we measured drag (Fx) and lift (Fz) across multiple orientations of a natural tumbleweed in a wind tunnel. These tests revealed that drag varied cyclically with orientation, particularly at higher wind speeds, while lift showed strong orientation dependence across all tested velocities (Fig. 2N, 2O). In the upright 0° configuration, lift was nearly zero, anchoring the tumbleweed to the ground and supporting stable rolling. At ±90°, lift turned negative, increasing downforce and pinning the structure, thus raising rolling resistance. In the inverted 180° orientation, lift increased sharply. This orientation-dependent force interplay, combined with the mechanical flexibility of the tumbleweed’s branches, underpins its unique rolling behavior (Supplementary Movie 1). At low wind speeds, drag-induced torque initiates stable rolling and eventual inversion. Once inverted, the flexible upper branches in contact with the ground elevate rolling resistance, creating a metastable, frustrated state. However, the increased lift in this position promotes destabilization, allowing the tumbleweed to return to its upright rolling state (Fig. 2P). At high wind speeds, the combination of a pronounced lift peak at 180° and elastic recoil from compressed branches propels the structure off the ground, resulting in hops. Hop amplitude scales with both wind speed and initial momentum. At approximately 12 m/s, large and irregular hops were observed, suggesting an evolved aerodynamic adaptation to aid long-range seed dispersal (Fig. 2Q). This sequence offers key insights into wind-driven tumbleweed mobility.

### Bio-inspired spheres - Characterization and mesh network implementation

**Fig. 3:**
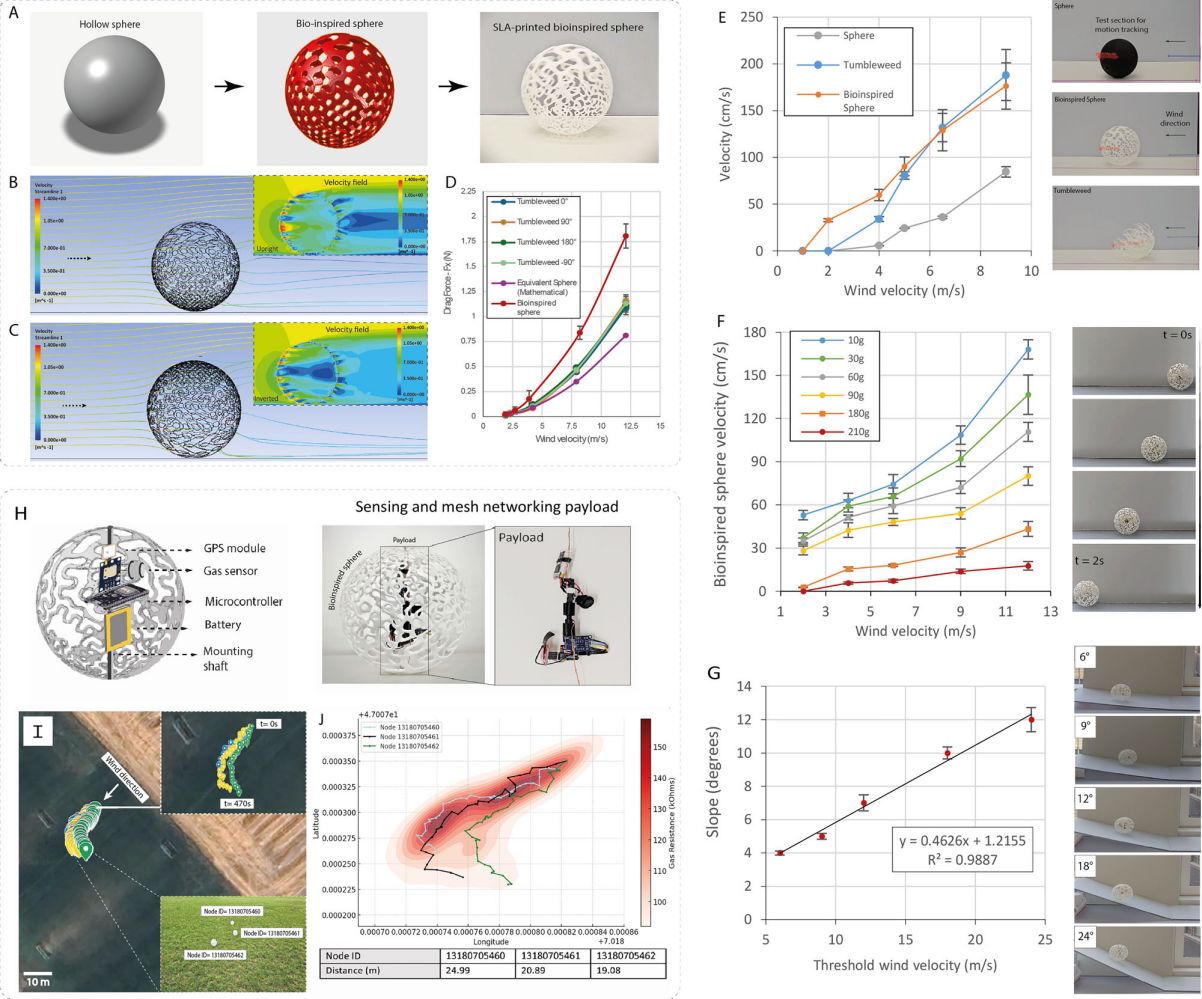
Passive mobility performance and environmental sensing field trials of HERMES. **A** Design progression from a solid sphere to a bio-inspired porous sphere **B, C**: CFD velocity fields comparing a solid sphere and porous sphere. **D** Drag force comparison across wind speeds for different designs. **E** Threshold wind velocity required to initiate motion (*n* = 3). Translational velocity vs. wind speed for solid sphere, natural tumbleweed, and bio-inspired sphere. **F** Load carrying capacity of the bio-inspired sphere and corresponding translation velocities at various wind speeds. **G** Slope climbing ability of a 50 g payload equipped bio-inspired sphere at various wind speeds. **H** Sensing payload design with GPS, microcontroller, battery, and gas sensor. **I** Field deployment showing node dispersal and GPS trajectories. **J** Sensor heatmap overlay illustrating distributed environmental mapping.

We translated the key aerodynamic insight, a vertical porosity gradient, into simplified hollow spheres via implicit geometry modeling (Fig.3A). Rather than mimic the intricate tumbleweed morphology, which complicates fabrication and payload integration, we designed a 1.5 mm-thick shell with a porosity gradient from top to bottom. The structure was fabricated via SLS, yielding a lightweight (~65 g) sphere with internal volume for payloads. This design aimed to retain tumbleweed-like mobility while addressing natural limitations such as rolling resistance from barbs and oblong shapes.

Wind tunnel tests confirmed the aerodynamic benefit of this design. At 12 m/s, the bio-inspired sphere generated 2.15 N of drag, compared to 1.1 N for natural tumbleweed C and 0.74 N for a solid sphere (Fig. 3D). CFD simulations revealed similar wake asymmetries and velocity fields, confirming that the porosity gradient exhibited drag-enhancing dynamics (Fig. 3B,C).

Mobility testing demonstrated clear performance advantages. The sphere began rolling at just 1 m/s wind speed, better than tumbleweed C (2 m/s) and solid spheres (3 m/s). At 6 m/s, it reached a speed of 1.5 m/s, tripling the solid sphere’s velocity (Fig. 3E). To assess payload capacity, we mounted central weights and recorded rolling velocities across wind speeds (Fig. 3F). At 4 m/s, the sphere carried 3.5× its own mass; at 12 m/s, it climbed 24° inclines with a 60 g load (Fig. 3G). Although capable of carrying up to 210 g, mobility degraded at high loads, suggesting an optimal payload of ~100 g for this geometry.

A portion of this capacity was allocated for electronics to form a mesh network of multiple tumbleweed-inspired rovers for environmental sensing. Each sphere integrated a BME688 gas sensor, Neo-6M GPS module, and Xiao ESP32C3 microcontroller, forming a Wi-Fi mesh network using the PainlessMesh protocol. This allowed each unit to serve as both node and relay, enabling real-time data sharing without centralized infrastructure^33,34^. The electronics were integrated within the sphere as shown in Fig. 3H. Powered by a 100 mAh battery, each node achieved 50 m communication range under line-of-sight conditions, with stable operation under a 10% duty cycle. While Wi-Fi was used for simplicity, long-range protocols could enable energy-efficient coverage over kilometers.

Field tests with three sensor-equipped spheres in open terrain (ambient wind ~8–14 km/h) demonstrated autonomous wind-driven dispersal over ~20 m within 8 minutes. Sensor data, including gas resistance and GPS coordinates, were relayed to a base station, enabling construction of spatial gas concentration maps (Fig. 3I, J). Terrain features modulated motion, slopes altered rolling resistance, and grass introduced irregular friction. The spheres halted when wind dropped below threshold, but sensing and transmission continued uninterrupted. While functional for short-term validation, the system’s operational life was limited (~1597 mAh/day). Future iterations will explore optimization strategies such as aggressive deep sleep cycles, low-power communication protocols, reduced packet sizes, and adaptive transmission intervals to extend mission duration. This short-range demonstration validates the fundamental mobility principles for future long-distance deployments.

### Active control implementation

While the bio-inspired spheres effectively collected environmental data when wind-driven, their dependence on ambient wind limits broader applicability. Calm conditions or complex terrain can hinder dispersal, highlighting the need for onboard propulsion. Fortunately, the sphere’s high payload capacity makes it well-suited for integrating active control mechanisms, enabling motion redirection or escape maneuvers when wind is insufficient for sustained or reliable locomotion.

**Fig. 4:**
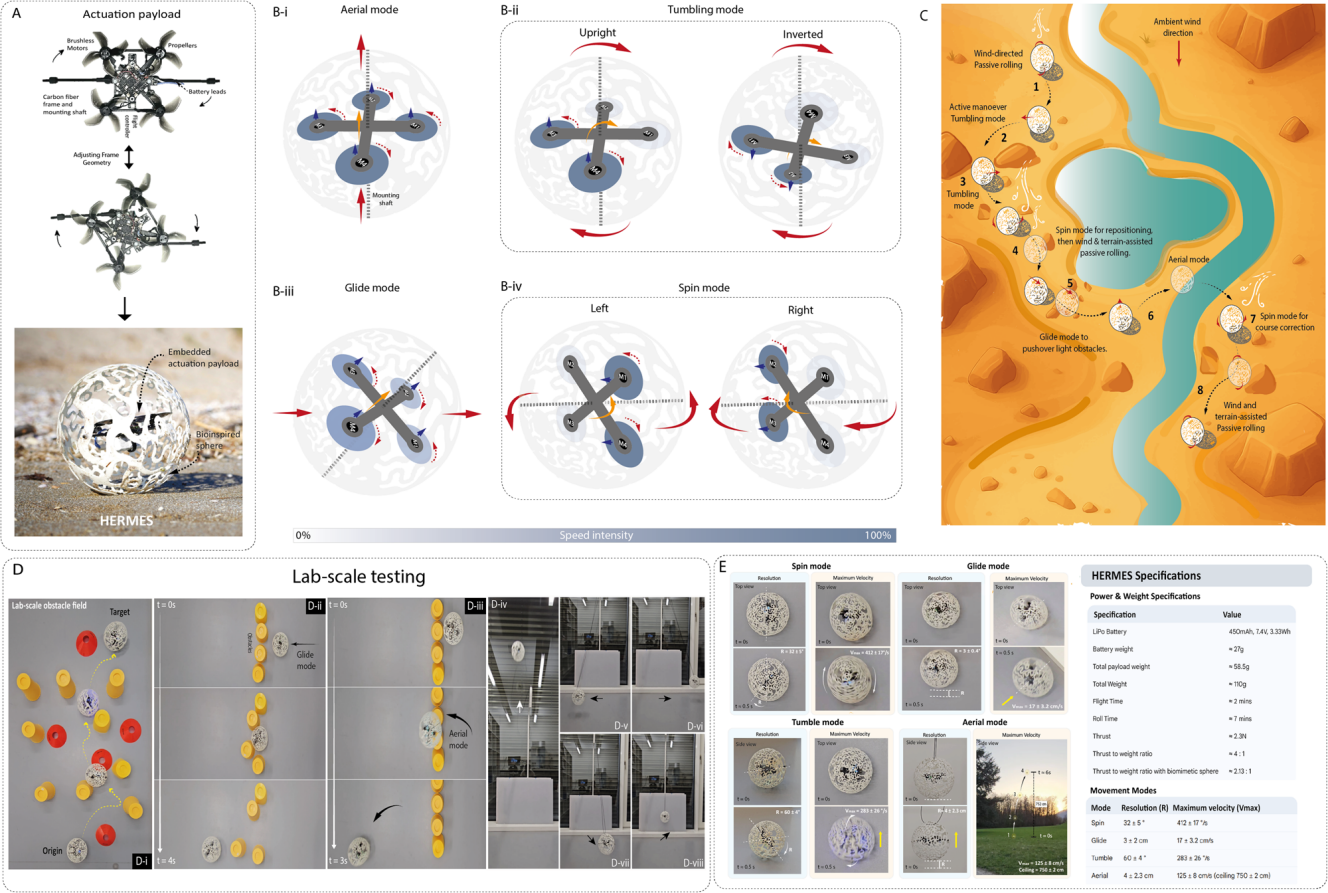
HERMES - Active locomotion characterization. **A** Quadrotor payload with integrated into the bio-inspired sphere to form HERMES. **B** Control strategies of the embedded quadrotor actuator: (B-i) aerial mode with full-thrust lift, (B-ii) tumbling mode using alternating diagonal motors, (B-iii) glide mode for short forward bursts with low thrust, (B-iv) spin mode using opposing motor pairs for yaw rotation, motor speed intensity bar (0–100%) shown. **C** Envisioned scenario showing sequential use of modes to navigate complex terrain (1), tumbling (2–3), spin reorientation (4), gliding (5), aerial lift (6), course correction (7), and return to passive motion (8). **D** Lab-scale demonstrations showcasing active navigation modes: (D-i) obstacle traversal, (D-ii) pushing over, (D-iii) flying over obstacles, (D-iv–viii) omnidirectional maneuverability during aerial mode. **E** Experimental results and the resulting specification sheet for each mode.

To enable controlled mobility under low-wind conditions, we embedded a lightweight quadcopter within the bio-inspired sphere (Fig. 4A) via rigid attachment. The quadcopter was chosen for its simplicity, multimodal navigation and ability to operate effectively in multiple orientations. When all motors engage, the system achieves full aerial mobility, while selective activation of motor pairs enables terrestrial modes such as tumbling, gliding, and spinning (Fig. 4B-ii–iv). Thanks to its spatial symmetry, thrust-vector flexibility, and elimination of gimbals or linkages, HERMES offers a simple and energy-efficient hybrid platform. The system achieves a thrust-to-weight ratio of 4:1 (2.13:1 with the shell) and is powered by a 450 mAh 2S battery, supporting ~2 minutes of flight and ~7 minutes of rolling (n = 3). CFD simulations confirmed that the porous shell induces backpressure and recirculation, reducing effective thrust (Supplementary Fig. 3A, B). Nonetheless, with pulse width modulation (PWM) control, four robust locomotion modes are possible, enabling adaptive navigation across diverse terrain as envisioned in Fig. 4C.

Tumbling Mode (Resolution: 60 ± 4°, Velocity: 283 ± 26 °/s, Energy: 11.1 mWh/s): When the quadcopter lies parallel to the ground in the upright orientation, the rear motors (M3–M4) are pulsed at maximum PWM, pitching the sphere forward and inverting the structure. Upon inversion, these same motors would unintentionally reverse thrust. To preserve forward motion, control is handed to M1–M2, now at the rear, spinning in reverse to maintain torque direction (Fig. 4B-ii). This alternating motor activation enables controlled flipping, useful for terrain reorientation and directional change.

Spin Mode (Resolution: 32 ± 5°, Velocity: 412 ± 17 °/s, Energy: 11.1 mWh/s): With the quadcopter oriented vertically, opposing lateral motors (e.g., M1–M3) spin in opposite directions, inducing a rapid yaw rotation (Fig. 4B-iii). This allows HERMES to spin in place, enabling self-alignment for subsequent movement.

Glide Mode (Resolution: 3 ± 0.4°, Velocity: 17 ± 3.2 cm/s, Energy: 11.7 mWh/s): In vertical orientation, front motors (M1–M2) are set at ~50% PWM, rear motors (M3–M4) at ~55%, creating a slight pitch and forward thrust (Fig. 4B-iv). This lets the robot glide along the ground, ideal for crossing flat surfaces or nudging light obstacles without lifting (Fig. 4D-ii).

Aerial Mode (Velocity: 125 ± 8 cm/s, Ceiling: 750 ± 2 cm, Energy: 22.2 mWh/s): All four motors are engaged at full PWM for vertical lift-off (Fig. 4D-iii–viii). This high-power mode lets HERMES hop over tall obstacles or terrain, used only when passive modes fail.

Figure 4D-i demonstrates how HERMES transitions between rolling, gliding and spinning to navigate a lab-scale maze. The effectiveness of this thrust-induced terrestrial movement and a detailed specifications sheet of these modes is shown in Fig. 4E, Supplementary Table S4 and Supplementary Movies 2. Values are mean ± SD from *n* = 3. Although not built for upwind travel, HERMES was tested under controlled lab conditions with headwinds ranging from 0 to 4.12 m/s and crosswinds from 0 to 3.8 m/s (Supplementary Notes S3, Fig. S4, Movie S4, S5). At 1.75–2.5 m/s—where passive rolling occurs and active compensation may be needed—HERMES maintained forward progress and positional retention at the lower end and exhibited controlled retracement at the higher end. Crosswind accuracy declined from ~97% to ~45%, with energy costs of ~11–26 mWh. Though energetically costly, brief upwind or crosswind maneuvers can support mesh- restoration and upwind sensing gradients. While the current system is tele-operated, future versions may enable autonomous mode switching via onboard IMU-based logic that detects stagnation or impacts. A four-state finite state machine illustrating this energy-aware control architecture is provided as a theoretical framework in Supplementary Fig. 5 and Notes S4 for future implementations.

### Hybrid Navigation and Field Deployment

**Fig. 5:**
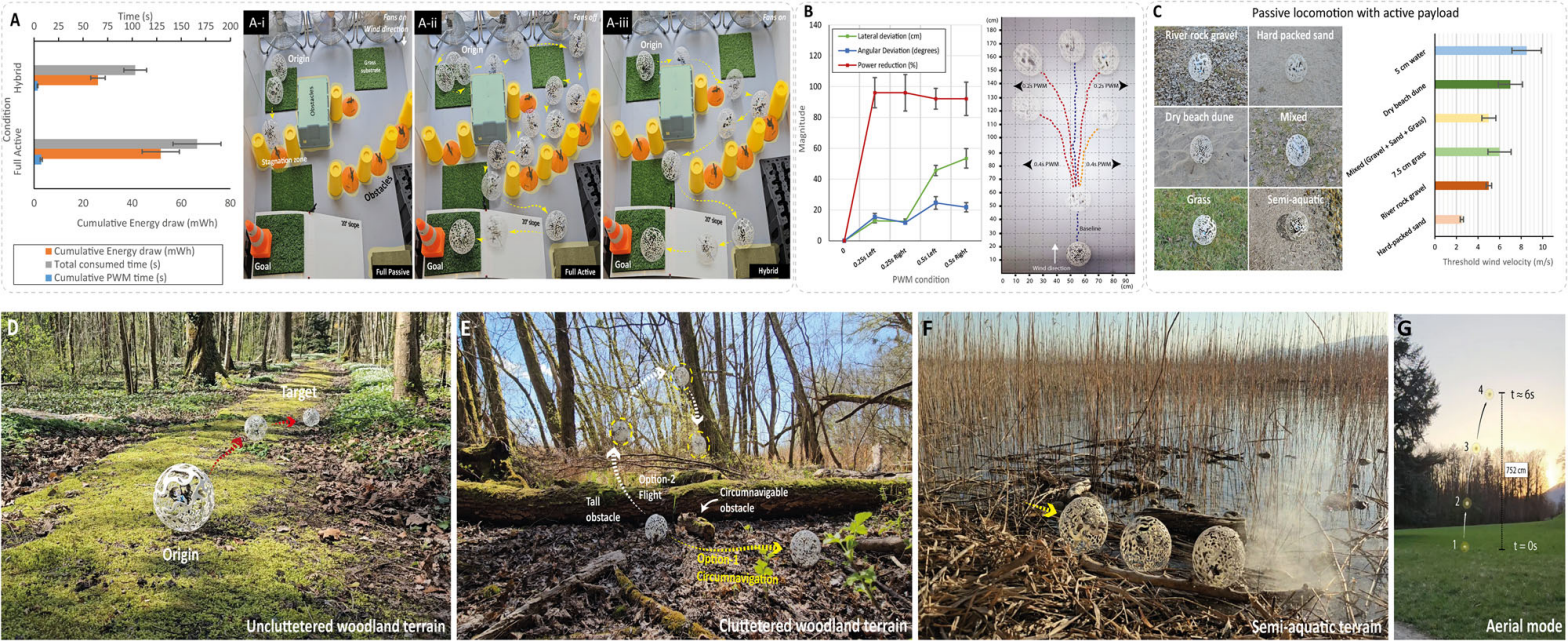
Hybrid navigation energy economy and field deployment of HERMES. **A** Cumulative energy draw, total time consumed, and cumulative PWM activation time for fully passive, fully active, and hybrid modes during maze navigation (**A-i, A-ii, A-iii)** Trajectories of passive, active, and hybrid navigation respectively. Data are expressed as mean ± standard deviation (SD), based on n  =  3 samples. **B** Hybrid control characterization at 5 m/s wind speed, showing lateral and angular deviation achieved with brief PWM bursts (0.25 s, 0.5 s), alongside power reduction values for the hybrid approach. **C** Threshold wind speeds required for passive locomotion of HERMES with embedded active payload across different terrains. **D** Active navigation in uncluttered woodland terrain. **E** Obstacle negotiation options in a cluttered woodland terrain: circumvent a smaller barrier with minimal actuation or fly over a tall obstacle **F:** Semi-aquatic terrain traversal with partial submersion ( ~ 5 cm depth) **G:** Real-world aerial mode testing demonstrating a ceiling altitude of 7.50 m.

To characterize energy use under different mobility conditions, we evaluated HERMES’s performance in an obstacle course subjected to wind speeds ranging from 6 m/s to 1 m/s, generated by three 150 W fans. Three conditions were tested: fully passive (Fig. 5A-i), fully active (Fig. 5A-ii), and hybrid navigation (Fig. 5A-iii). In the passive case, HERMES quickly stagnated due to obstacles. In the fully active case, it completed the course successfully but at a high energetic cost ( ~ 50 mWh) and took ~166 s. In contrast, the hybrid condition, which combined wind propulsion with brief PWM thrust bursts, enabled faster completion ( ~ 105 s) with just ~26 mWh of energy, demonstrating a 48% improvement in energy efficiency and 37%-time savings over active-only control (Fig. 5A). Notably, developed airflow within the maze guided HERMES along favorable paths which, in active-only mode, required multiple PWM bursts for correction.

To test control efficiency, we conducted hybrid course correction experiments (Fig. 5B). Short PWM bursts enabled angular adjustments of ~25–50°, resulting in lateral shifts of ~15–50 cm depending on motor pair. This minimal intervention approach reduced energy consumption by ~90–95% relative to continuous actuation. When coupled with algorithms that predict entrapment, such targeted corrections can prevent energy-intensive recovery attempts and extend the operational range of wind-driven robots in complex terrain.

Finally, HERMES was tested in natural outdoor settings to assess real-world locomotion. Despite the added mass of the quadcopter payload, the robot retained passive mobility across varied terrain types (Fig. 5C, Supplementary Movie 3). CFD simulations confirmed the quadcopter module had negligible aerodynamic impact during rolling (Supplementary Figure 3C, D). Wind thresholds ranged from ~2 m/s on hard soil to ~7.5 m/s on grassy terrain, confirming minimal passive mobility loss. We then demonstrated fully active locomotion without wind across varied terrains. In open woodland (Fig. 5D), tumbling enabled direct traversal; in cluttered terrain (Fig. 5E), HERMES bypassed obstacles via gliding and flight. In semi-aquatic settings (Fig. 5F), thrust-assisted movement enabled partial submersion over ~5 cm water. In full aerial mode, it reached 7.5 m altitude (Fig. 5G), clearing major obstacles. These results highlight the feasibility of combining passive and active mobility for real-world exploration.

## Discussion

By transforming drag into an ally, we demonstrate that wind can drive terrestrial passive devices, when mimicking the porosity gradient observed in tumbleweeds. Our wind tunnel experiments and CFD simulations confirm that this structural asymmetry amplifies pressure drag and generates distinct wake profiles in upright and inverted orientations. These effects enable stable rolling and increased payload capacity. While earlier wind-driven concepts enlarged cross-sectional area, creating bulky structures, we show that reconfiguring void space through porosity distribution offers a more scalable and efficient alternative.

Prior studies on porous bodies established that low-porosity structures behave like solids, moderate porosity reduces drag, and high porosity can increase drag via near-surface turbulence^35–38^. Our 2D CFD-based drag model shows that porosity asymmetry critically influences aerodynamic performance. A strong contrast between upper and lower hemisphere porosities produces asymmetric wake separation and increased drag. Based on a simplified 5 mm substructure, the model offers a quantitative framework for tuning drag via porosity gradients. Future refinements may involve variable substructure size or branching geometry. Combined with branch elasticity and lift effects, the vertical porosity gradient governs orientation-dependent locomotion modes such as somersaulting, frustrated states, and hopping, resulting in a distinct and repeatable mobility behavior.

We applied these principles to 3D-printed hollow spheres for enhanced passive mobility. They carried 3.5× their weight at 4 m/s wind, climbed 24° slopes with a 60 g load, and outperformed tumbleweeds and solid spheres. Embedded sensors enabled wind-driven dispersal and data transmission across 50 m in open terrain. These results validate the potential of passive sensor swarms dispersed by wind. Future versions could use long-range protocols like LoRa^39^ or satellite IoT^40^ for continent-scale coverage. Techniques such as visual odometry^41^, inertial navigation^42^, received-signal-strength localization^43^ or distributed aerial relays^44^ could extend functionality to GPS-denied environments. Beyond gas sensing, the payload bay may support cameras, or other sensors of exobiological or environmental interest.

To address terrain entrapment and unreliable wind, we integrated a quadcopter into the porous sphere, creating HERMES, a hybrid system that rolls passively but activates tumbling, gliding, spinning, or aerial escape only when needed. Maze experiments showed HERMES reduced energy consumption by 48% and traversal time by 37% compared to fully active motion. HERMES lies at the intersection of passive mobility, drone–rover hybrids, and active rolling mechanisms. Unlike traditional hybrids, it defaults to passive locomotion and relies on minimal motor bursts, achieving hybrid mobility, escape capabilities, and superior payload-to-weight ratios beyond those of passive systems (Supplementary Tables S1–S3). However, several limitations remain. Our current field demonstrations validate short-range mobility, serving as proof-of-concept for the underlying hybrid locomotion principles. While natural tumbleweeds can disperse over hundreds of kilometers, scaling to such extensive ranges would require substantial advances in power systems, autonomous navigation, and long-term durability that remain as future work. Current demonstrations focus on short-range, proof-of-concept validation with teleoperated active control. However, an autonomous control framework using IMU-based finite state machine logic could enable future autonomous operation (Supplementary Fig. S5). Autonomy at the current stage remains theoretical and represents a key area for development in this work. The 2-minute hover time enables obstacle avoidance but limits sustained flight, especially on harsh terrains like Martian regolith or polar ice^45^. Future efforts will explore swarm coordination, adaptive clustering, and field validation^5,46–55^. On worlds where sunlight is scarce and wind is abundant, robots that roll passively and fly when needed offer a compelling path for exploration.

To conclude, on worlds where sunlight is scarce and wind is abundant, tumbleweed-like robots that roll passively and fly occasionally, offer an attractive route for terrestrial exploration. By rolling into the unknown with wind as an ally and thrust as a judicious reserve, HERMES thus sets the stage for hybrid robotic explorers.

## Methods

### Natural tumbleweed selection and characterization

The tumbleweeds used in this study were sourced from commercially available plants, and no living plants were harmed in the collection process. All necessary precautions were taken to handle the plants ethically and responsibly. Tumbleweed C, selected from a group of tumbleweeds (labeled A-F) purchased from West Texas, was chosen for further study based on its relatively lightweight structure and optimal circularity, which were determined through visual inspection and a series of measurements. The tumbleweed was CT-scanned to create a 3D digital model, which was then repaired and optimized for 3D printing. The resulting model closely mimicked the geometry of the real tumbleweed. The CT scan and optimization process were performed using the RX-SOLUTIONS Ultratom CT system and Amira Avizo v 2022.2 software to generate a high-quality STL file suitable for 3D printing.

### 3D printing and model fabrication

The 3D printing of the tumbleweed model was done using Selective Laser Sintering (SLS) using an EOS P395 printer and PA2200 material to replicate the complex, delicate geometry of the real tumbleweed. PA2200 was chosen due to its strength and durability, which were necessary for the robust testing in the wind tunnel and mobility tests. The printed prototype was compared to the original tumbleweed in terms of shape and structural integrity to ensure that it could withstand wind-driven motion without the fragility of the real specimen.

### Wind tunnel testing

Drag forces measured in the wind tunnel were compared between the tumbleweed prototype, the bio-inspired sphere, and the solid sphere at the HEPIA wind tunnel facility (Geneva), while the flow visualizations were carried out in a custom-made wind tunnel (0.9 m long, 0.5 m wide test section, and 8.5% blockage ratio) at 1 m/s wind velocity. The results were analyzed to quantify the differences in drag generation, and the lift forces (Fz) for the upright and inverted configurations were compared. CFD results were validated against the wind tunnel data to ensure accuracy. The mobility data for each object were determined on flat ground with a wind source, PrimaClima PK160TC.

Wind tunnel experiments were performed to measure the drag forces generated by the tumbleweed prototype (tumbleweed specimen C) in different orientations (upright, inverted, and several intermediate configurations). The wind tunnel was set to a range of wind speeds (1–12 m/s) to simulate different environmental conditions. For each configuration, the drag force was recorded using a RUAG multi-component balance mounted to the model. Custom jigs were used to orient the model in different configurations to measure orientation-dependent aerodynamic forces. To compare the results, an equivalent solid sphere was also tested under identical conditions. Flow visualization was performed using smoke at 1 m/s to observe the wake patterns and turbulence generated by the tumbleweed in both upright and inverted configurations.

### Computational Fluid Dynamics (CFD) simulations

Computational fluid dynamics (CFD) simulations were conducted using a combination of Ansys Fluent and COMSOL Multiphysics. 2D CFD simulations were performed on the middle slice of the real tumbleweed geometry, obtained from the CT scan data, in both upright and inverted configurations. These simulations aimed to replicate the flow profiles observed in the wind tunnel. A porosity gradient ranging from 80% at the top to 60% at the bottom of the tumbleweed was integrated into the CFD model to study the effect of porosity on the aerodynamic behavior and drag generation. The flow field, including wake formation, pressure distribution, and flow separation, was analyzed to gain insights into the tumbleweed’s unique aerodynamic properties.

### Mobility testing

Mobility tests were conducted by measuring the object velocity of both the tumbleweed prototype and a bio-inspired sphere under varying wind speeds (1–6 m/s). The bio-inspired sphere was designed with a porosity gradient similar to that of the tumbleweed and was compared to a solid sphere to assess the differences in mobility. Object velocities were recorded using a camera and tracked using video modeling software - Kinovea-2023 by Joan Charmant and Physlets Tracker by OpenSource Physics, to determine the threshold wind speed required for movement and the maximum velocities achievable at higher wind speeds.

### Active navigation payload

HERMES incorporated a centrally integrated aerial and rolling propulsion system realized via an F4 2–3S 20 A All-in-One (AIO) flight controller with embedded DShot ESC protocol, controlled using the ExpressLRS protocol through a Radiomaster Zorro M2 radio controller. The flight controller was flashed with Betaflight firmware (v4.4.1) and bound to the transmitter using ExpressLRS at 2.4 GHz and the motor control and flight dynamics were configured in the Betaflight Configurator.

To enable the terrestrial modes, “Flip Over After Crash” window was repurposed to drive differential thrust-based rolling. A dedicated switch on the RC (mapped to AUX2) was assigned to toggle terrestrial modes via the Modes tab. In this mode, Betaflight restricts active motor output to two opposing motors spinning in reverse, allowing the system to roll forward or pivot without taking off. Bi-directional DShot and motor direction reversal were configured using BLHeli32, enabling quick motor direction changes essential for terrestrial maneuverability. Normal aerial mode was restored by switching out of terrestrial mode, reactivating synchronous motor operation for thrust-based flight.

The quadcopter used four 1102 18,000KV brushless motors with 4-blade propellers made of polycarbonate-nylon blend, mounted to a carbon fiber X-frame housed within a lightweight bio-inspired spherical cage. The propellers were oriented toward the plane of highest porosity in the sphere to reduce shell backpressure and flux confinement. All components were powered by a 2S 450 mAh 75 C LiPo battery mounted at the system’s center of mass to preserve balance. Further, the Master Multiplier in the PID tuning window was altered to identify ideal settings, and a value of 0.9 offered stable control in both aerial and terrestrial modes. Together, the quadcopter payload weighed approximately 60 g, and through a force gauge, it was found to produce 2.3 N of thrust, resulting in a thrust-to-weight ratio of 4:1.

This configuration allowed seamless teleoperation for switching between mobility modes: (1) passive wind-driven rolling, (2) active rolling via differential motor thrust, and (3) aerial repositioning using thrust-based flight, thereby enabling robust multimodal exploration over diverse terrain types. In the future, autonomous mobility with IMU-based logic will be explored.

### Mesh network integration

A Wi-Fi mesh network was implemented using the PainlessMesh library ^33^on XIAO ESP32C3 microcontrollers, enabling decentralized, real-time communication. Each bio-inspired sphere was equipped with a BME688 gas sensor for environmental monitoring and a Neo-6M GPS module for location tracking. Its combined weight with 100 mAh battery was 28 g. With a duty cycle of 10% (data transmitted every 10 s), it had a power draw of ~1597 mAh/day. Sensor data, including gas resistance (kΩ) and GPS coordinates, were transmitted through the mesh network, allowing seamless data sharing across multiple units. The system ensured position-agnostic sensing, with each robot acting as both a transmitter and receiver. Logged data were analyzed for network range, transmission reliability, and swarm coordination efficiency.

### Data analysis

Statistical Analysis - All quantitative experimental values were presented as mean ± standard deviation (SD) of the mean from *n* = 3.

## Supplementary information

Supplementary Information

Description of Additional Supplementary Files

Supplementary Movie 1

Supplementary Movie 2

Supplementary Movie 3

Supplementary Movie 4

Supplementary Movie 5

Transparent Peer Review file

## Data Availability

All data corresponding to the figures in the main text and supplementary are available here.
