# Supplementary material for: Tumbleweed-inspired robots with hybrid mobility for terrestrial exploration
Source: Nat Commun. 2025 Nov 20;16:11519. doi: 10.1038/s41467-025-66513-1 (PMC12749897; doi:10.1038/s41467-025-66513-1)
Supplement: Supplementary file 1 — Supplementary Information [file 41467_2025_66513_MOESM1_ESM.pdf]

SUPPLEMENTARY INFORMATION

Tumbleweed-inspired robots with hybrid mobility for terrestrial exploration

Sanjay Manoharan [1]*, Biruktait Lemecho [1], Mustafa M. Fadlelmula [1] and Vivek Subramanian [1]*

[1] Laboratory for Advanced Fabrication Technologies, Institute of Electrical and Micro Engineering, École Polytechnique Fédérale de Lausanne (EPFL), Neuchâtel 2000, Switzerland

This file contains -
Supplementary movies S1-3; Supplementary table -S1, S2, S3 and S4 Supplementary figures S1-S4 along with corresponding supplementary notes S1 and S2

Supplementary table-S1: Comparison of HERMES with contemporary hybrid and multimodal robotic platforms.

Platform	Mass (kg)	Mobility Modes	Peak Speed	Payload	Adaptability	Autonomy Level	Highlights
**HERMES** *(Our work)*	0.11	Wind-roll, Thrust assisted roll or flight	1.5 m s^{-1} roll; 0.125 m s^{-1} hover	~30g gas sensor + GPS + WiFi mesh in passive mode	Grass, rocks and partial water submersion (5 cm)	Autonomous passive mode; Teleoperated active mode	Ultralight hybrid; sub-second mode switch
**AirCrab**[29]	2.65	Quadcopter -hover + perch-wheel	~0.3 m s^{-1} ground perch	Light micro-gripper	Indoor perching only	Teleoperated: ground manipulator requires human-in-loop	Beam-gripping micro-drone
**Rollocopter**[28]	-	Hexacopter -hover + sphere roll	Sim. 4.0 m s^{-1} roll	-	Concept only (sim only)	Planned autonomous: energy-aware simulated	Omnidirectional cage rolling
**M4 Morphobot**[47]	6.0	Quadcopter -flight, wheeled, legged	2.3 m s^{-1} drive	~1 kg	Tumble, WAIR climb	Semi-autonomous: waypoint tracking + manual morph triggers	8 mobility modes
**LEONARDO (LEO)**[26]	2.58	Walk + thrust-balance	1.4 m s^{-1} walk; 6 m s^{-1} flight	None stated	Slackline, stairs	Teleoperated: balance and movement manually triggered	Lift-aided biped tricks
**AgriBot**[27]	-	Quadcopter -hover + skid-rover	~0.4 m s^{-1} rover	Sprayer/plucker	Crop fields, canals	Semi-autonomous: depth-trigger pop-up to flight	Field-sensing and terrain switching
**GOAT**[48]	2.8	4-wheel drive roll and swim; No flight	3.8 m s^{-1} roll; 2.4 m s^{-1} drive	1.6 kg	Snow, river, rocks	Semi-autonomous: GNSS rover drive + passive roll; manual re-morph	Morph-driven terrain transitions; low-energy descent
**FSTAR**[22]	0.9	Quadcopter -hover + sprawl-enabled wheeled run	2.6 m/s crawl; 2.82 m/s transition speed	-	Pipes, indoor obstacles, outdoor terrain	Autonomous straight-line run; Teleoperated transitions	Reconfigurable sprawl geometry; crawl/fly with same motors; 20:1 wheel gearing

Supplementary table-S2: Detailed Technical Comparison with Flight/ Rolling Mechanisms

Reference	Flight via	Rolling via	Weight	Payload/ Payload-weight ratio	Points to consider
**Dudley et al., 2015 - Aerial terrestrial robot (ATR)**[49]	Standard quadcopter	Tilting quadcopter creates thrust component to independently rotate the exoskeleton via axle-bearings	10.36 g	~5g / ~0.48:1	Passive railcar-like ring geometry + bearing + yaw imbalance. Limited 10.36g payload. Requires careful pitch-angle control to avoid lift-off. Turning depends on ring diameter contact and friction. Bearing introduces drag and complexity; friction varies over time. No glide or spin-in-place mode; transitions less flexible. No wind-assisted locomotion capability.

**Kalantari et al., 2014 (HyTAQ)** [25]	Standard quadcopter	Cage rolls via freely rotating revolute joints at arm mounts	~600g	N/A	All motors must run continuously even for ground movement. No active control over rolling direction; limited terrain adaptability. Relies entirely on propeller thrust for movement. Poor maneuverability; exposed propellers; bearing friction losses. No passive wind-driven mode - purely energy-consumptive.
**Briod et al., 2014 (Gimball)** [50]	Standard quadcopter	No rolling	~370g	~250-300g / ~0.81:1	Focus on collision tolerance only, no terrestrial mobility. Single-mode operation limits energy efficiency. No ground-based navigation capability.
**Salaan et al., 2019**[51]	Standard quadcopter	Hemispherical passive wheels on each rotor spin due to rotor thrust	N/A, expected to be heavier based on dimensions	250-300g / ~0.16:1	Ground mobility via passive wheels lacks dedicated actuators. Gimbal mechanism adds mechanical complexity and failure risk. Limited directional control on ground. No wind-assisted locomotion. Continuous motor operation required for any movement.
**Atay et al., 2020**[52]	Conceptual	Conceptual	Theory only	Theory only	Theoretical analysis only - no physical implementation or validation. Cannot assess real-world performance limitations.
**Kalantari et al., 2020 (Drivocopter)**[53]	Standard quadcopter	Dedicated motors drive Actuated Propeller Cages (APCs)	5,000 g	~1,000g / ~0.2:1	Heavy (5kg); increased complexity and weight due to extra powered wheel actuators. High energy consumption from dual actuation systems. No passive locomotion modes.
**Jia et al., 2023 (Quadrolltor)**	Standard quadcopter with folding arms	Yaw torque induces rolling (body and cage coupled)	350g	N/A	Rolling depends on yaw torque; limited turning sharpness and speed. Mechanical complexity due to passive joints, spring-loaded mechanisms and joint stoppers. No autonomous navigation. No wind-assisted capability. Poor rolling precision (32.3° RMSE).
**Bhardwaj et al., 2024 (ATOM)**[54]	Self-rotating mono-wing (clockwise/counterclockwise)	Differential thrust generates torque for rolling	~124g	No payload testing	Flight stability difficult; limited flight control; must stop before sharp turns. Short 4.8min flight time. Transition success rate only 95%. Max 2 m/s ground speed. High power consumption during stops/turns. Wing stall at low RPM. No passive locomotion modes.
**HERMES** *(Our work)*	Standard quadcopter with a flight controller to permit thrust reversal	4 distinct modes: Tumbling (283°/s), Spin (412°/s), Glide (17 cm/s), + Passive wind-driven	~120g	210g in passive mode/1.75:1 Active flight based on thrust ratio (theory) =139g /1.15:1	Primary passive operation (zero power most of time). 48% energy improvement via hybrid control. 90-95% power reduction through selective actuation. Multi-terrain capability (2-7.5 m/s wind thresholds). Semi-aquatic operation. No mechanical complexity - same actuators for all modes. 7.5m flight altitude capability.

Supplementary table-S3: HERMES vs Bio-Inspired Passive Robotic Systems

**Capability**	**Iyer et al. (Dandelion-inspired, *Nature 2022*)[3]**	**Light-controlled seeds (Dandelion-inspired, *Advanced Science 2023*)[4]**	**3D Electronic Microfliers (inspired by various wind dispersed seeds, *Nature 2021*)[55]**	**Solar-powered Origami (leaf-falling inspired, Science Robotics 2023)[56]**	**NASA Polar rover (2004)[16]**	**HERMES** *(Our work)*
**Total Weight**	30mg	1.2mg	Various: mg-scale	414mg	~20kg	**123g**
**Payload Capacity**	Tiny sensors (GPS, radio)	Minimal	Electronics + sensors	Microcontroller, Bluetooth, sensors	~5kg	**210g passive, 52-139g active**
**Locomotion Modes**	1 (passive only)	1 (passive + light shape change)	1 (passive only)	2 (tumbling + stable descent)	1 (passive wind only)	**6 total (4 active + 2 passive)**
**Active Control**	None	Terminal velocity modulation via light	None	Shape change via solar-powered actuator	None	**Full aerial + terrestrial locomotion**
**Escape Capability**	Cannot escape stagnation	Cannot escape stagnation	Cannot escape stagnation	Cannot escape stagnation	Cannot escape stagnation	**Active escape via tumbling/flying**
**Obstacle Navigation**	None	None	None	None	Roll over only	**Active bypass + climbing**
**Range Extension**	At mercy of wind	At mercy of wind	At mercy of wind	Limited shape control	At mercy of wind	**Hybrid wind + active control**
**Mission Flexibility**	Single deployment	Single deployment	Single deployment	Limited programmable behavior	Single deployment	**Multi-modal adaptive missions**
**Energy Management**	Solar + battery-free	Passive only	Battery-free wireless	Solar-powered actuation	Passive only	**Hybrid 48% energy savings**
**Deployment Scale**	50-100m range	Lab demonstration	Not specified	98m in light breeze	131km (until wind stopped)	**Scalable with active repositioning**
**Mission Continuation**	Ends when wind stops	Ends when wind stops	Ends when wind stops	Ends when wind stops	Permanently stopped in Greenland	**Continues via active modes**
**Communication Range**	N/A	None	Wireless capability	60m Bluetooth	Satellite (global)	**Scalable with mesh network**
**Key Features**	95% upright landing	80 Hz flapping capability	3D bio-inspired structures	Mid-air shape transitions	Real-world 131km traverse	**Hybrid passive-active paradigm**

Supplementary Table S4. HERMES specifications under laboratory testing conditions.

**Specification**	**Value**
LiPo Battery	450 mAh, 7.4 V, 3.33 Wh
Battery weight	≈ 27 g
Total payload weight	≈ 58.5 g
Total Weight	≈ 110 g
Flight Time	≈ 2 mins
Roll Time	≈ 7 mins
Maximum flight speed	125 ± 8 cm/s
Maximum altitude	≈7.5 m
Glide mode speed	17 ± 3.2 cm/s
Aerial mode power	22.2 mWh/s
Terrestrial modes power	11.1-11.7 mWh/s
Thrust	≈ 2.3 N
Thrust to weight ratio	≈ 4 : 1
Thrust to weight ratio with biomimetic sphere	≈ 2.13 : 1

Supplementary notes

S1. Impracticality of Inflatable Spheres for Rolling Locomotion

While inflatable spheres may seem like a potential solution for mimicking tumbleweed-like rolling locomotion, calculations reveal their impracticality for compact robotic swarms. The aerodynamic drag force on an inflated sphere is far too low for effective wind-driven movement, requiring an unreasonably large cross-sectional area to achieve the necessary forces for locomotion. For instance, a sphere would need a wind velocity of at least 13 m/s to overcome rolling resistance, demanding a mass under 50g with a cross-sectional area of 0.6 m^2, impractical for compact robots. Achieving mobility in a compact form factor would require exceptionally high drag coefficients (see Supplementary), which remains impractical for compact designs.

Given this observation, we are thus motivated to find means of enhancing drag within practicable geometric constraints. Given the significant mobility demonstrated by natural tumbleweed, we begin by understanding drag in analogous geometries.

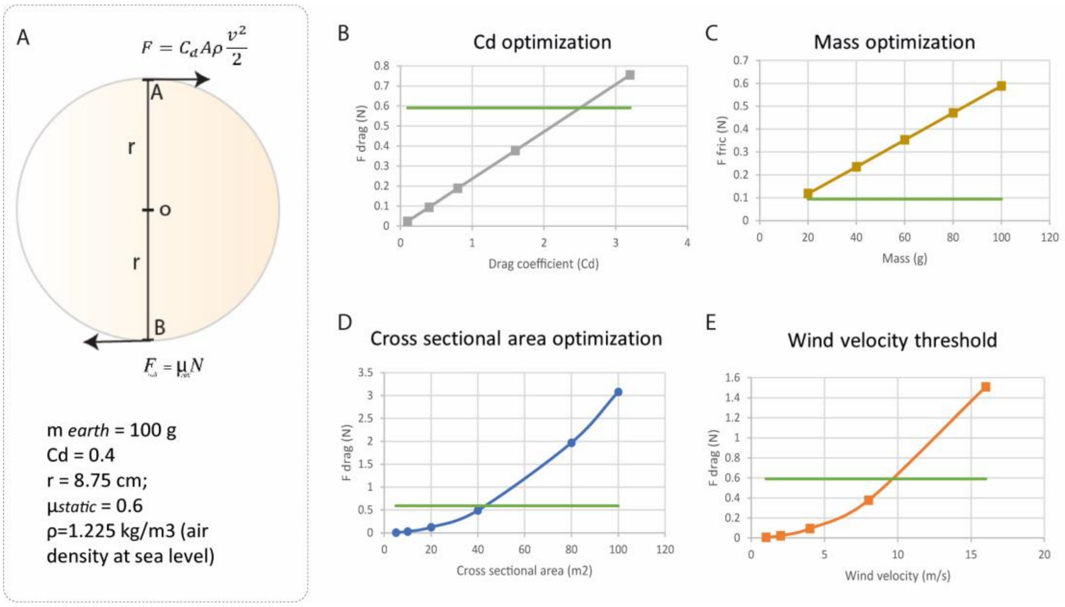

Supplementary Figure 1: Impracticality of Inflatable Spheres for Rolling Locomotion **A:** Wind-induced rolling requires drag force to exceed static friction **B–E:** Parametric analyses show that realistic values of drag coefficient, mass, area, and wind speed are insufficient for initiating motion in compact inflatable spheres. For example, achieving locomotion requires wind speeds >13 m/s, cross-sectional areas >0.6 m^2, or masses <50 g. These impractical requirements highlight the need for alternative geometries to enhance drag, as observed in natural tumbleweeds.

S2. Refined Drag Model

To quantify how porosity gradients affect drag in tumbleweed-inspired rolling, we developed a refined drag model based on 2D computational fluid dynamics (CFD) simulations (Fig. 3A).

The porosity gradient is defined as $\Delta p = pt - pb$, where pt and pb represent the porosity at the top and bottom hemispheres, respectively. The baseline drag for a solid sphere is given by:

$$D_{sphere} = \frac{1}{2} \rho A v^2 C_{d0}$$

where ρ = air density, A = frontal area, v = velocity, and C_{d0} = drag coefficient.

To correct for porosity effects, we simulated porous circles with substructure radius of 5 mm and Δp values ranging from –0.11 to +0.11 across Reynolds numbers of 50,000 to 1,000,000. Results revealed a parabolic relationship between drag and Δp, especially at higher Re, confirming that velocity amplifies the aerodynamic impact of porosity distribution. This led to a refined drag coefficient expression:

$$C_{d(\Delta p)} = a_0 + a_1 \Delta p + a_2 (\Delta p)^2,$$

where $\{a_0, a_1, a_2\}$ are regression coefficients obtained from simulations.

The results show a parabolic relationship between drag and porosity gradient (Fig. 3B), with stronger effects at higher Reynolds numbers,indicating that drag sensitivity increases with velocity. When $pt > pb$, increased top porosity alters wake separation; when $pt < pb$, bottom porosity affects stability; and when $pt \approx pb$, drag is minimized due to symmetric airflow. This model thus highlights the critical role of porosity gradients in enhancing drag and provides guidelines for optimizing stability and control in porous designs.

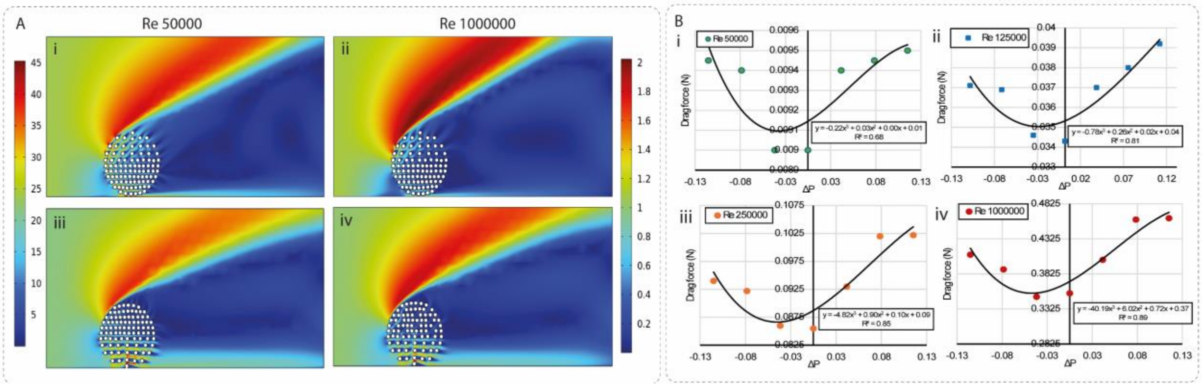

Supplementary Figure 2. Effect of Porosity Gradient (Δp) on Drag Force Across Different Reynolds Numbers.

A: Flow field visualizations from 2D CFD simulations at Re = 50,000 (i, iii) and Re = 1,000,000 (ii, iv) for porous circular structures with higher top porosity (i, ii, Δp =0.11) and higher bottom porosity (iii, iv, Δp = -0.11). Velocity contours align with tumbleweed flow patterns and drag forces from structures with Δp between 0.11 to -0.11 at various Reynolds numbers were used for B. **B**: Parabolic dependence of drag force on Δp across four Reynolds numbers: (i) 50,000, (ii) 125,000, (iii) 250,000, and (iv) 1,000,000. Polynomial regression shows stronger porosity effects at higher Re, while lower Re exhibits weaker correlation due to reduced wake asymmetry.

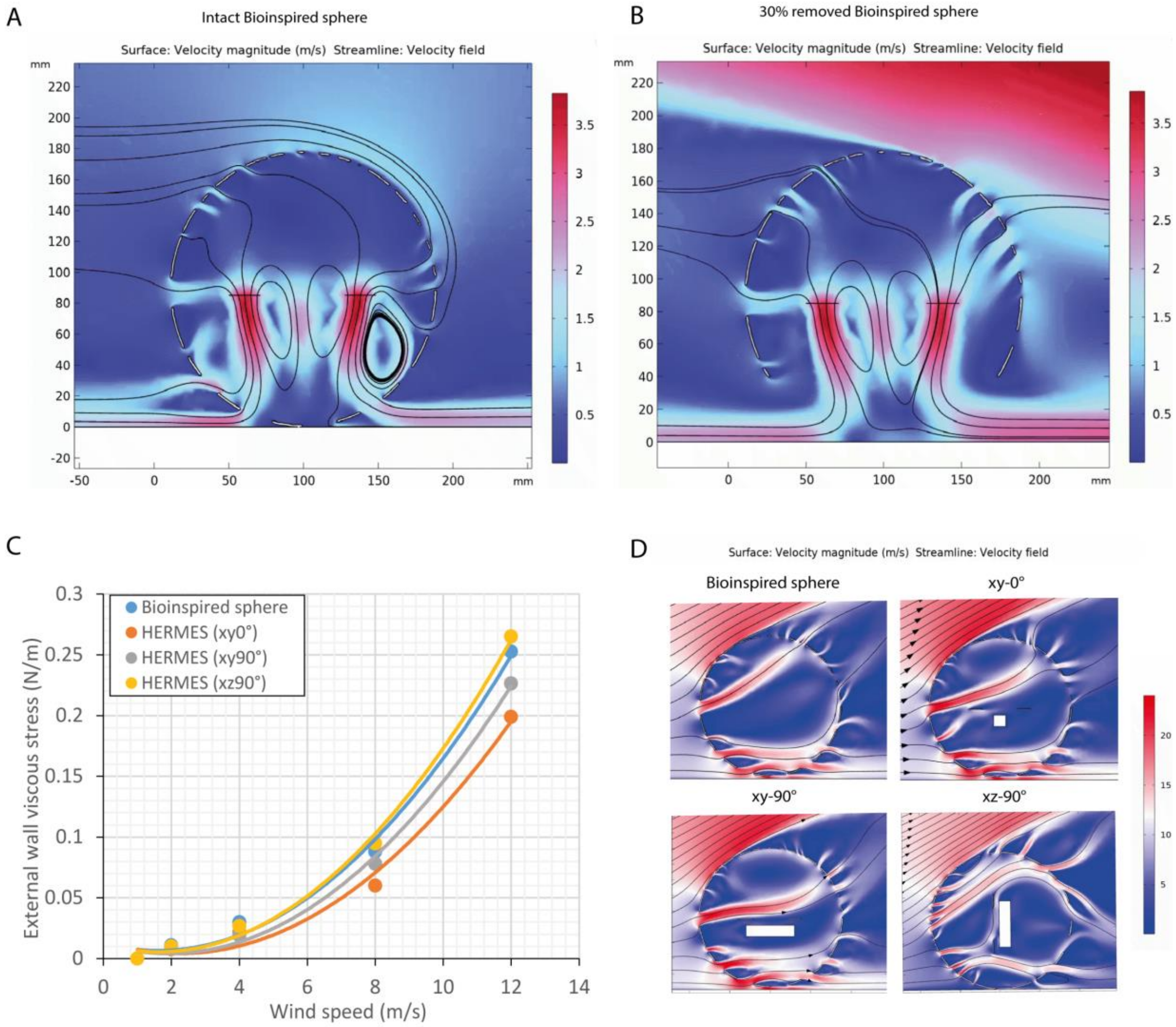

Supplementary Figure 3: Aerodynamic confinement and the effect of quadcopter payload on the drag of the bio-inspired sphere for passive rolling
A: CFD simulation of internal propeller flow within an intact bio-inspired sphere shows strong recirculation and limited thrust escape due to the enclosing porous shell. **B:** CFD simulation with 30% of the lower shell removed reveals increased upward flux escape and reduced flow confinement. **C,D:** Influence of an active payload on drag in the intact sphere. C: External wall viscous stress as a function of wind speed for the bio-inspired sphere and the HERMES robot in three orientations (xy-0°, xy-90°, xz-90°), showing drag sensitivity to payload geometry and alignment. D: Corresponding 2D CFD visualizations illustrate how changes in orientation alter the exposed cross-section of the internal quadcopter, resulting in minimal flow impedance in xy-0° and xy-90°, and maximal impedance in xz-90°. Overall, the embedded payload only marginally affects surface shear, indicating that passive wind-driven locomotion is largely preserved in the hybrid HERMES configuration.

S3: Active Mobility in Adverse Wind Conditions

Although not designed for upwind or crosswind travel, short upwind transits, though energetically costly, can be valuable for escaping terrain traps, re-establishing mesh connectivity, countering gust-induced drift, or reaching upwind sensing gradients. To address concerns regarding energy costs in unfavorable wind directions, both perpendicular crosswind and parallel headwind experiments were conducted using a controlled laboratory setup with a fixed test section and variable fan-generated wind speeds.

Headwind Performance: For headwind characterization, the robot was positioned against a solid block to prevent passive backward drift prior to actuation, and wind speeds were systematically varied from 0 to 4.12 m/s. At each condition, HERMES was activated with 0.5 s PWM bursts to execute tumble-mode locomotion, while forward displacement was tracked using video analysis (Supplementary Fig. 4A). Results revealed three distinct operational regimes: (1) At wind speeds ≤1.75 m/s, HERMES maintained effective forward locomotion with minimal degradation, achieving ~75% of its no-wind displacement. (2) At intermediate speeds (2.1–2.5 m/s), the system exhibited controlled retracement behavior, advancing before reversing toward its origin, indicating preserved directional control rather than chaotic drift. (3) At higher wind speeds (3.75–4.12 m/s), forward progress was minimal, but active positioning remained functional. During upwind traversal under moderate drag, HERMES transitioned from tumbling to a transient spinning gait before reversal, marking a distinct stall-spin-retracement sequence (Supplementary Fig. 4C).To quantify headwind performance, we employed 1D position error analysis appropriate for forward displacement assessment:

Position Error = $|x_{target} - x_{actual}|$

Accuracy = 1 - (Position Error / Intended distance)

Effective Energy Cost = Total energy / Accuracy

Using the 0 m/s condition (95.2 cm forward) as the intended target, effective energy costs increased from 5.57 ± 0.13 mWh at baseline to 19.21 ± 1.73 mWh at 4.12 m/s headwind, representing a 245% energy penalty (Supplementary Fig. 4B). We introduce the return coefficient R = dnet/dmax, where dnet is the final net displacement from origin and dmax is the maximum forward displacement achieved during the trial. HERMES displayed R=1 at 0 m/s, declining to R=–0.57 at 2.5 m/s and remaining negative at higher speeds, capturing the transition from traversal to stable retracement.

Crosswind Performance: Using an identical experimental setup with fans positioned 90° to the intended motion direction, crosswind experiments were conducted across wind speeds ranging from 0 to 3.8 m/s. HERMES was positioned against a blocking surface and activated using standardized 0.5 s PWM tumbling actuation, while motion tracking analysis captured lateral drift patterns, forward progress efficiency, and directional stability (Supplementary Fig. 4D).For crosswind analysis, we employed 2D position error methodology accounting for both forward progress and lateral positioning accuracy:

Position Error = $\sqrt{[(x_{target} - x_{actual})^2 + (y_{target} - y_{actual})^2]}$

Accuracy = 1 - (Position Error / Intended distance)

Effective Energy Cost = Total energy / Accuracy

Using the 0 m/s endpoint (129.7, 5.8 cm) as the intended target, crosswind conditions showed progressive but manageable performance degradation (Supplementary Fig. 4E). At low crosswinds (≤1.8 m/s), HERMES maintained excellent accuracy (97 ± 1.5%) with minimal energy penalty (11.4 ± 0.2 mWh). Moderate crosswinds (2.25–2.85 m/s) reduced accuracy to 56-58% with effective energy costs of 19.2-19.8 mWh. High crosswinds (3.2–3.8 m/s) showed continued forward motion despite significant lateral drift, maintaining 36-49% accuracy with energy costs reaching 30.5 ± 9.7 mWh at maximum crosswind—representing a 669% energy penalty for maintaining spatial targeting precision.

Results demonstrated progressive but manageable performance degradation with increasing crosswind velocity. Forward progress ranged from 129.7 ± 1.2 cm at baseline to 99.8 ± 15.5 cm at 3.8 m/s crosswind, while lateral drift increased from 5.8 ± 0.5 cm to -71.2 ± 15.0 cm, indicating controlled but significant positional deviation under strong crosswind conditions.

Notably, no catastrophic failures were observed across either wind condition tested (Supplementary Fig. 4C,D). The PWM-based active control system remained responsive in all scenarios, validating the hybrid passive–active strategy under challenging wind conditions. These comprehensive results demonstrate that HERMES retains functional locomotion across all tested wind directions, with graceful performance degradation rather than operational failure, directly addressing energy cost concerns under diverse atmospheric conditions.

Interestingly, the reversal trajectories in upwind conditions (Supplementary Fig. 4A) and lateral drift patterns in crosswind conditions (Supplementary Fig. 4D) followed predictable arc-like paths, where trajectory curvature can potentially be tuned via actuation timing and angle of attack relative to the wind vector. This suggests that controlled drift behavior could serve as a functional locomotion mode for lateral repositioning or wind-guided reorientation in future implementations. To conclude, at wind speeds between 1.75 and 2.5 m/s—which reflect typical conditions on laboratory flooring where passive motion is feasible but active compensation may be needed, HERMES demonstrates effective forward locomotion and stable positioning at the lower end of the range, and controlled retracement with a return coefficient of $R = -0.57$ at the higher end. Under crosswind conditions, positional accuracy remains high (~97%) at 1.75 m/s but drops to ~45% at 2.5 m/s, with corresponding energy expenditures ranging from approximately 11 to 26 mWh. The combined headwind and crosswind characterization validates HERMES's operational robustness under unfavourable conditions, providing quantified energy-accuracy trade-offs essential for mission planning in variable atmospheric environments.

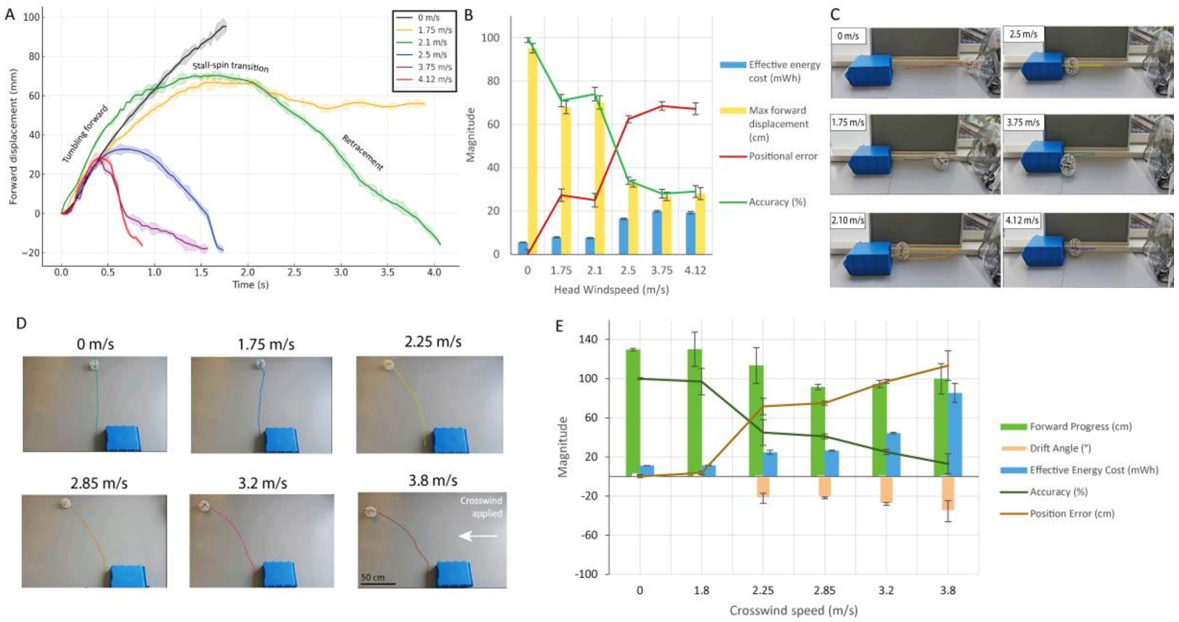

Supplementary Figure 4: Headwind and crosswind performance characterization of HERMES under controlled wind conditions. A: Forward displacement trajectories during upwind travel reveal three distinct behavioural regimes with increasing headwind speed. **B:** Quantification of positional accuracy, energy cost, and forward displacement under headwinds. **C:** Side-view snapshots of robot trajectories under varying headwind intensities. **D:** Top-view trajectory traces under crosswind perturbation (0–3.8 m/s), illustrating lateral drift behavior across trials. **E:** Crosswind metrics including forward progress, drift angle, energy cost, accuracy, and position error. Data are shown as mean ± SD (n = 3). Results validate HERMES's resilience to lateral and opposing wind vectors, demonstrating maintained control and progressive performance degradation across tested conditions.

S4. Autonomous Control Implementation for HERMES

Energy-Escalation State Machine Design

HERMES in its current state only possesses an onboard IMU, without additional sensors for positional or environmental awareness. This limits the feasibility of implementing sophisticated autonomy algorithms. To navigate this constraint, we propose an IMU-only control logic built on a hierarchical finite state machine that escalates energy use progressively, resorting to higher-cost interventions only when lower-energetic cost strategies fail. This may be implemented in future and can preserve the energy-efficient philosophy of the platform, while enabling autonomous stagnation recovery and obstacle navigation.

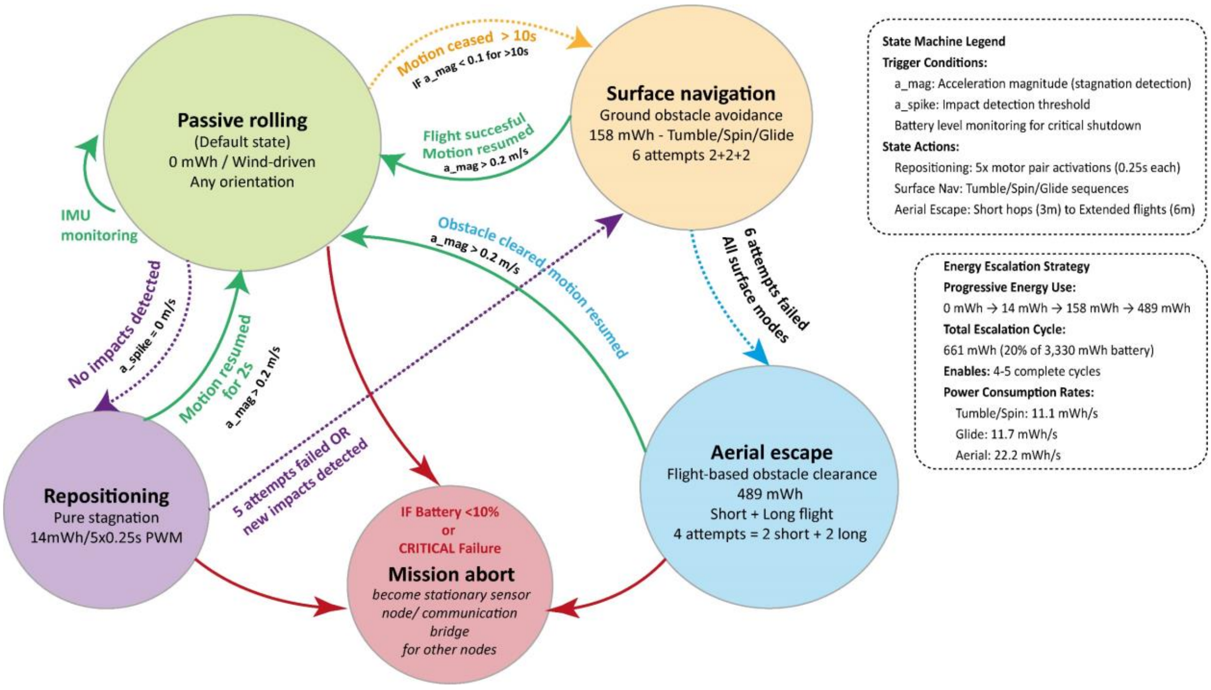

Supplementary Figure 5: Autonomous energy-escalation control logic for hybrid locomotion in HERMES. Finite-state diagram showing how HERMES escalates from passive wind-driven rolling to increasingly active modes based on IMU data. Stagnation without impacts triggers low-power Repositioning. Impacts or failed attempts lead to Surface navigation (158 mWh) via spin, tumble, and glide. Persistent failure escalates to Aerial escape (489 mWh) using short and long flights. Success is defined as sustained motion via accelerometer data. Critical failure or low battery triggers Mission abort and safe shutdown.

Four-State Autonomous Architecture

Passive Rolling (Default State - 0 mWh) The robot operates in wind-driven mode with zero active energy consumption, leveraging ambient airflow for locomotion. IMU sensors continuously monitor acceleration magnitude (a_mag) and impact signatures (a_spike) to detect state transition triggers. The spherical geometry enables stable rolling in any orientation, eliminating the need for active reorientation common in traditional mobile robots.

Repositioning (Minimal Intervention - 14 mWh) Triggered when motion ceases (a_mag < 0.1 m/s² for >10s) without impact detection, indicating pure stagnation rather than obstacle collision. The system executes five sequential 0.25-second motor pair activations using different combinations (M1-M2, M3-M4, M1-M3, M2-M4, random) to test orientation changes for wind capture. Each attempt consumes approximately 2.8 mWh, totaling 14 mWh for the complete sequence.

Surface Navigation (Ground-Based Obstacle Avoidance - 158 mWh) Activated by impact detection (a_spike > 3 m/s²) or repositioning failure. The system employs IMU orientation data to select energy-optimal locomotion modes: when the mounting shaft is perpendicular to

ground, spin mode (11.1 mWh/s) is prioritized; when parallel, tumbling mode is used first. The complete sequence includes 2 attempts each of tumbling, spinning, and gliding modes, consuming 66.6 + 33.3 + 58.5 mWh respectively.

Aerial Escape (High-Energy Flight - 489 mWh) Reserved for scenarios where surface navigation fails completely. Progressive escalation includes short hops (4s duration, 50-100cm altitude) followed by extended flights (7s duration, 2-7.5m altitude). All four motors operate at 70-100% PWM, consuming 22.2 mWh/s during active flight phases.

Energy Budget Analysis

With a 3,330 mWh battery capacity (450 mAh × 7.4V), the worst-case complete escalation cycle consumes 661 mWh (approximately 20% of total capacity). This enables 4-5 full escalation cycles before mission termination, while typical operations requiring only repositioning or surface navigation consume <5% of available energy. The energy allocation reflects measured consumption rates: tumbling and spinning operations consume 11.1 mWh/s, gliding requires 1plot mWh/s, and aerial modes demand 22.2 mWh/s. These empirically-derived values enable precise energy budgeting for autonomous mission planning.

Failure Recovery and Adaptive Strategies

Rather than immediate mission termination, HERMES can implement intelligent failure classification and degraded operation modes. Mobility failures trigger transition to stationary sensor node operation, maintaining environmental monitoring capabilities while conserving energy for periodic escape attempts if paired with a energy harvester. Extended dormancy protocols maybe used for long-term hibernation with daily or weekly repositioning attempts during favourable wind conditions. Battery-critical scenarios can prioritize data preservation and mesh network relay functions, allowing immobilized units to serve as communication bridges for active swarm members.

Future improvements

HERMES could enhance autonomy while preserving energy efficiency through lightweight sensor integration and intelligent control. Low-power sensors like ultrasonic or thermal anemometers (<2 mWh) could enable predictive obstacle avoidance and airflow sensing, while barometers may inform wind-aware repositioning. Gyroscope-accelerometer fusion could improve terrain detection and motion classification. Machine learning on IMU data may enable adaptive thresholds and mode selection, and AI-based vibration analysis could distinguish between soft and hard obstacles. Event-triggered vision and swarm map-sharing could further reduce navigation overhead. Energy harvesting via flexible photovoltaics, piezoelectrics during tumbling, and regenerative braking during flight could extend mission life. These upgrades preserve the passive-first philosophy, using high-energy modes only when necessary.
