# Supplementary material for: Tumbleweed-inspired robots with hybrid mobility for terrestrial exploration
Source: Nat Commun. 2025 Nov 20;16:11519. doi: 10.1038/s41467-025-66513-1 (PMC12749897; doi:10.1038/s41467-025-66513-1)
Supplement: Supplementary file 2 — Description of Additional Supplementary Files [file 41467_2025_66513_MOESM2_ESM.pdf]

Description of Additional Supplementary Files

Supplementary movie 1: Locomotion mechanism of natural tumbleweeds Lab-scale testing of natural tumbleweeds illustrating the various locomotion modes depending on low or high windspeeds. High and moderate windspeeds exhibited a hopping behaviour while low windspeeds led to stable rolling

Supplementary movie 2: HERMES active navigation - Laboratory tests Laboratory demonstration of HERMES showcasing its omnidirectional surface and aerial navigation possibilities for obstacle avoidance or wind-less conditions

Supplementary movie 3: HERMES Active and passive navigation - Field tests Real-world tests highlighting the hybrid navigation possibilities for various terrain and active locomotion strategies for overcoming obstacles of various scales and environments

Supplementary movie 4: Headwind performance analysis via motion tracking Demonstrating HERMES's forward displacement under varying headwind intensities under controlled lab conditions. The robot initiates active tumbling via a 0.5 s PWM pulse revealing the threshold wind speeds beyond which passive resistance dominates and reversal occurs.

Supplementary movie 5: Crosswind performance analysis via motion tracking Demonstrating HERMES's directional persistence under crosswinds (0-3.8 m/s) with standardized 0.5s PWM tumble from a fixed starting position. Motion analysis quantifies lateral drift versus forward progress across increasing crosswind velocities
